# The use of food swaps to encourage healthier online food choices: a randomized controlled trial

**DOI:** 10.1186/s12966-021-01222-8

**Published:** 2021-12-04

**Authors:** Laura Jansen, Ellen van Kleef, Ellen J. Van Loo

**Affiliations:** Marketing and Consumer Behaviour Group, Wageningen University & Research, Hollandseweg 1, 6706 KN Wageningen, the Netherlands

**Keywords:** Swap offer, Nutri-Score, Descriptive norm message, Situational motivation, Healthy food choices

## Abstract

**Background:**

Online grocery stores offer opportunities to encourage healthier food choices at the moment that consumers place a product of their choice in their basket. This study assessed the effect of a swap offer, Nutri-Score labeling, and a descriptive norm message on the nutrient profiling (NP) score of food choices in an online food basket. Additionally explored was whether these interventions made it more motivating and easier for consumers to select healthier foods and whether potential effects were moderated by consumer health interest.

**Methods:**

Hypotheses were tested with a randomized controlled trial (RCT) in a simulated online supermarket. Dutch participants (*n* = 550) chose their preferred product out of six product options for four different categories (breakfast cereals, crackers, pizza, and muesli bars). Participants were randomly allocated to one of eight groups based on the interventions in a 2 (Nutri-Score: present, not present) X 2 (swap offer: present, not present) X 2 (norm message: present, not present) between subject design. The primary outcome was the difference in combined NP score of product choices, for which a lower score represented a healthier product.

**Results:**

Swap offer (B = − 9.58, 95% CI: [− 12.026; − 7.132], Ƞ^2^ = 0.098) and Nutri-Score labeling (B = − 3.28, 95% CI: [− 5.724; −.829], Ƞ^2^ = 0.013) significantly improved the combined NP score compared to the control condition (NP score M = 18.03, SD = 14.02), whereas a norm message did not have a significant effect (B = − 1.378, 95% CI [− 3.825; 1.070], Ƞ^2^ = 0.002). No evidence was found that interventions made it more motivating or easier for consumers to select healthier food, but situational motivation significantly influenced the healthiness score of food choices for both swap offer (b = − 3.40, *p* < .001) and Nutri-Score (b = − 3.25, *p* < .001). Consumer health interest only significantly moderated the influence of Nutri-Score on ease of identifying the healthy food option (b = .23, *p* = .04).

**Conclusions:**

Swap offer and Nutri-Score labeling were effective in enhancing healthy purchase behavior in the online store environment.

**Trial registration:**

This study was retrospectively registered in the ISRCTN database on 02-09-2021 (ISRCTN80519674).

**Supplementary Information:**

The online version contains supplementary material available at 10.1186/s12966-021-01222-8.

## Introduction

Digital interventions to promote healthier food choices in the online environment have attracted a lot of interest recently [1]. An important way to encourage consumers to consider other options in an online choice environment is through the use of a digital recommender system [2]. A digital recommender system offering consumers the possibility to swap their initial food choices with similar product alternatives - called a swap offer - has been used to encourage consumers to make healthier food choices [3, 4]. Examples of food swaps aiming at healthier food purchases are a swap product lower in salt [3, 5], energy density [6], or saturated fat [7, 8]. Such interventions have proven to successfully change food-purchasing behavior in both physical (e.g. [4, 9, 10]) and online environments (e.g. [3, 4, 7]), as they can help people in expending their competencies of choosing healthy food by asking them to think about the risk of their unhealthy choice in a transparent and understandable form [11].

The use of nutrition labels is a common way to help consumers evaluate the healthfulness of products. Front-of-pack (FOP) nutrition labels are used to provide information to consumers in more understandable formats to encourage healthy consumption [12]. A variety of FOP nutrition labels have emerged, of which Nutri-Score labeling is found to be one of the most promising labeling systems to help consumers identify and rank nutritonal quality of food [13–15]. Nutri-Score is “a graphic scale that divides the nutritional score into five classes (expressed by a color and a letter), the purpose being to help the consumer better see, interpret and understand the nutritional quality” ([16], p.24]). It is a summary indicator label, meaning that it gives an overall rating of product healthfulness [12]. Nutri-Score was developed by Santé Publique France [16] and has been implemented in several countries such as France, Belgium, Spain, the Netherlands, and Germany, and is also likely to be implemented in other countries in the near future to inform consumers about the healthfulness of products [17, 18]. What little research has performed on Nutri-Score showed a positive significant effect of Nutri-Score on understanding nutritional quality of purchases (e.g., [18–20]). However, previous research mainly showed only small improvements in the nutritional quality of real-life supermarket food purchases [21] and opted to combine Nutri-Score labels with other interventions, such as educational leaflets, to improve the effectiveness of Nutri-Score [22].

Aside from providing tools to consumers to make healthy food choices, it is also expected that a descriptive norm message, which is a norm about what the majority of people do [23], can guide consumers toward healthier choices. Social norms are considered one of the most effective ways to nudge people toward a certain behavior [24] and have proven to successfully promote healthy eating behavior (e.g., [25, 26]). Socials norm messages are effective because people want to obtain social approval from others and be judged positively [25, 27]. The current study explores the effect of providing a swap offer, Nutri-Score label, or descriptive norm message on the nutrient profiling (NP) score of food choices, for which a better (lower) NP score is expected (hypothesis 1). Additionally, the combined effect of all three strategies seems fruitful to investigate, as they may strengthen each other.

Additionally, we explore whether ease of identifying the healthy food option and situational motivation to choose healthily are mediators between the effect of swap offers, Nutri-Score labelling, and norm message on NP score. For ease of identification, a swap offer is expected to have a positive effect because it increases the visibility of a healthy product (hypothesis 2). Consumers receive feedback that the chosen product is not the healthiest option and are offered a healthier alternative instead. Regarding Nutri-Score, nutrition labels that are easier to interpret seem to have an effect on the ability to identify healthier food choices due to the simplicity of the color and grading system (hypothesis 4) [19, 28–30]. Clear and precise labeling is preferred over ambiguous statements because it increases consumers’ perceived control and ability to choose or reject a certain product [31].

For the mediator situational motivation, which is defined as motivation experienced during a particular activity [32], the shopping task, swap offer, Nutri-Score label, and norm message are expected to increase situational motivation because presence of the interventions increases involvement with the product [33]. Being presented with a healthier alternative makes consumers aware of the (un)healthfulness of their food choice which subsequently may motivate them to behave more healthily (hypothesis 3) [34]. Additionally, presence of and attention to the Nutri-Score label make healthfulness information more salient, which may remind consumers about healthy eating and thus motivate them to make a healthy choice in this particular shopping situation (hypothesis 5) [34]. Descriptive social norms have the ability to influence behavior because people want to follow others’ behavior to behave efficiently [23]. The logic of “if everyone is doing it, it must be a sensible thing to do” [35, p.1015] serves as a time- and effort-saving shortcut that leads to a certain behavior. Hence, a message with a descriptive norm referring to what peer groups often do can motivate consumers to choose the healthier food option (hypothesis 6) (e.g., [36, 37]).

The proposed mediators are expected to have a positive effect on the NP score of food choices. Situational motivation leads to more healthful choices (hypothesis 7), because motivated people are more likely to invest effort in understanding provided product information and to make product choices accordingly [38, 39]. This means that people with high motivation are more likely to process provided information at product selection (i.e., Nutri-Score, swap offer or descriptive norm message) that guides consumers to healthier food choices. Additionally, healthfulness of choices is influenced by the ease of identifying the healthy food option (hypothesis 8). This because ease of identifying the healthy food options due to interventions such as Nutri-Score and swap offer increases the confidence of a person that (s)he can choose healthily, which leads to performing and maintaining healthy behavior [40]. Moreover, it is suggested that situational motivation moderates the effect of ease of identification on healthfulness of food choices (hypothesis 9) because the ability to perform a behavior most likely leads to performance only if the consumers also feel motivated [41]. In addition, people with high motivation and high ability are more likely to use all available product information for decision-making, whereas people with high ability but low motivation do not [39].

Finally, it is proposed that the effect of swap offer, Nutri-Score labeling, and norm message on ease of identification (hypothesis 10) and situational motivation (hypothesis 11) may depend on the consumers’ health interest. Interest to engage in health-related behavior can influence the willingness to process healthfulness information and therefore increase the positive effect of interventions [42, 43]. Additionally, if a person is highly interested in health-related behavior, an intervention leads to even more motivation than for those with low health interests because of the personal involvement [44, 45].

**Fig. 1:**
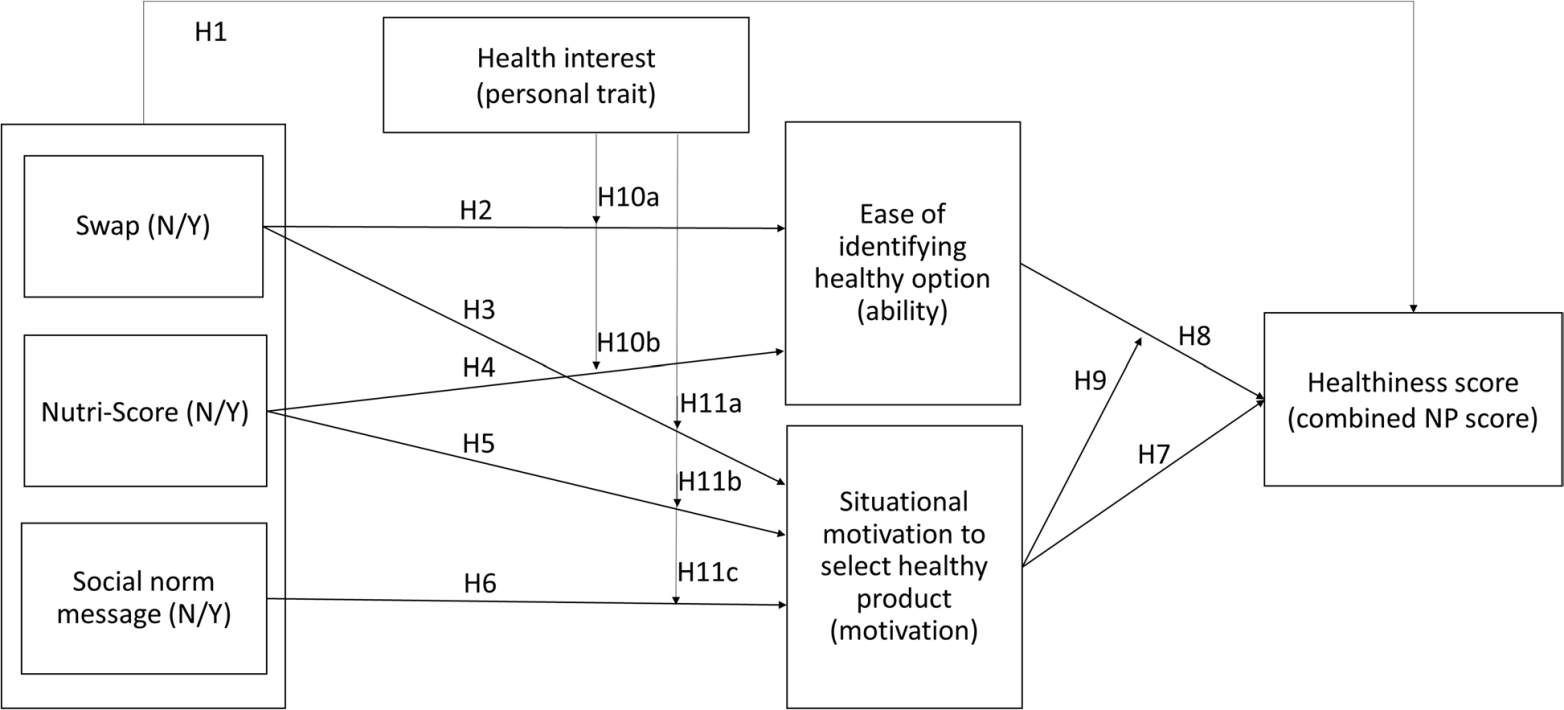
Conceptual framework

Hence, the main aim of this study is to investigate the effect of swap offer, Nutri-Score labeling, and norm message in an online store environment in order to evaluate whether these strategies can guide consumers toward healthier food choices. Additionally, we explore whether these interventions increase the ease of identifying healthier foods as well as the situational motivation to choose healthily, which is the motivation to choose in the here and now. Finally, we examine whether potential effects are moderated by consumers’ health interest. Hypotheses are presented in Fig. 1. Reporting is performed using the CONSORT checklist (Additional file 1), and a TIDier checklist for the interventions is provided (Additional file 2).

## Methods

### Study design

#### Study design

To test the hypotheses, a randomized controlled trial (RCT) was conducted in a simulated online supermarket where participants were evenly allocated to eight conditions in a 2 (swap offer: yes versus no) × 2 (Nutri-Score: yes versus no) × 2 (descriptive norm message: yes versus no) between subject design. At a simulated point of purchase of an online supermarket, each respondent individually was exposed to one of the eight conditions where swap offer, Nutri-Score label, and a descriptive norm message were present or not (Table 1).

**Table 1:** Overview of treatment conditions

Treatment	Nutri-Score	Swap offer	Norm message
1: Control (no intervention)	No	No	No
2: Nutri-Score	Yes	No	No
3: Norm message	No	No	Yes
4: Swap offer	No	Yes	No
5: Nutri-Score + norm message	Yes	No	Yes
6: Nutri-Score + swap offer	Yes	Yes	No
7: Norm message + swap offer	No	Yes	Yes
8: Nutri-Score + norm message + swap offer	Yes	Yes	Yes

#### The products in the simulated online supermarket

Within the Qualtrics survey, participants were shown a product assortment of four different product categories: breakfast cereals, muesli bars, crackers, and pizza (Additional file 3). Each product category consisted of six products with different NP score levels based on Nutri-Score. Participants were exposed to one of the four product categories at a time and were asked to indicate their choice. Both order of product categories and of the products within one assortment were randomized to prevent order bias. Brand and price information were deleted from all product images to prevent an effect from brand and/or price. An overview of the product assortment for each of the four categories is given in Additional file 3.

#### Nutri-score intervention

The effect of Nutri-Score was tested by displaying a Nutri-Score label alongside products in intervention conditions. Nutri-Score label was based on the nutrient profiling (NP) system of the UK Food Standards Agency [46], which uses a scoring system to determine nutrient content per 100 g of food or drinks [13]. To calculate Nutri-Score, positive and negative points are given to components of the food product. The final Nutri-Score is calculated by subtracting the total number of advantageous points from the total number of disadvantageous points. This way, a score between − 15 (healthy) and + 40 (unhealthy) is calculated. The healthiest choice is visualized with an A (dark green), and the lowest nutritional value is indicated with an E (red). For the calculation of the NP scores and the corresponding Nutri-Score, the method by Santé Publique France [16] was followed. An overview of NP scores of the used products, and its calculation is given in Additional file 4. The label was placed on the right side of product images in accordance with the online store environment of Delhaize [47]. An example of a question with a Nutri-Score label is given in Additional file 5.

#### Swap offer intervention

If the original chosen product was not the healthiest option in the assortment, a swap offer was provided with a healthier alternative. The product with the lowest NP score for each category was identified as the healthier alternative and used as a swap offer (see Additional file 3). If participants already chose the healthiest option, no swap was offered. In swap interventions with the Nutri-Score and/or the descriptive norm, the swap offer was displayed with the Nutri-Score label and/or a descriptive norm message (see Additional file 5), respectively.

#### Descriptive norm message intervention

The following descriptive norm was developed for the current study: *“Dutch consumers more often choose healthy products.”* This norm was based on results from the Dutch National Institute for Public Health and the Environment (RIVM) [48] showing that more and more Dutch consumers eat healthier. To increase the salience of the norm [23, 49], the text was presented in a green banner above the product options (see Additional file 5).

#### Exposure in each treatment group

Participants were exposed to one of the eight conditions (Additional file 6), and all participants were exposed to the same products (Additional file 3). Based on their treatment group (Additional file 6), the Nutri-Score, swap offer, and/or norm message were presented. Examples of product choices for each intervention condition are provided in Additional file 5.

### Participants

**Fig. 2:**
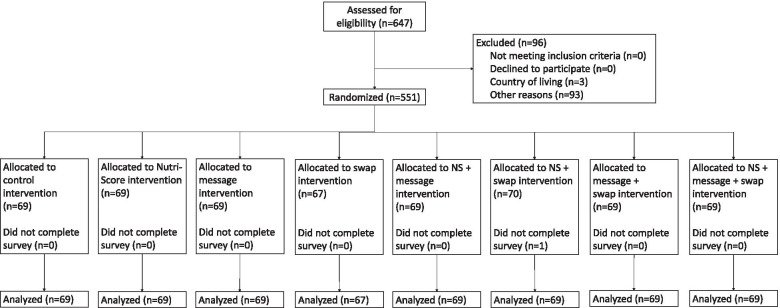
Flow diagram of the progress through the phases of a randomized controlled trial of eight groups

Participants were recruited by a market agency using a representative sample for the Netherlands in terms of age and gender (age and gender breakdowns from CBS [50]). Participants were eligible if they lived in the Netherlands, were older than 18, were able to read Dutch, and were willing to provide information. The survey language was Dutch. The required sample size was calculated with a power of 0.8 and an alpha of 0.05 (G*Power version 3.1.9.4) to detect a small effect size (partial Ƞ2 = 0.04 with corresponding effect size f = 0.2). This power analysis was performed for our primary hypothesis 1, meaning that the other hypotheses in our framework were explored without further analysis for power calculation. Based on this calculation, a total sample size of 472 participants was required. A final sample size of 550 participants was set to oversample each condition with approximately 10 participants. As such, the trial ended after 550 participants completed the full survey. In total, 647 people participated, of which 550 completed the full survey in May 2020. There was only one dropout in this study (allocated to Nutri-Score with swap offer intervention). Participants excluded after the demographics questions were non-Dutch (3 participants) or male (93 participants), since only Dutch participants were eligible, and we aimed for a representative sample in terms of age and gender. The flow diagram of participant allocation is given in Fig. 2.

### Measures

#### Dependent variable

The dependent variable *combined NP score* was the summated NP score of the chosen products of each of the four categories (NP scores for each product are given in Additional file 4). The summated NP score ranged from − 6 to + 58. The lower the score, the better the product healthfulness.

#### Mediators

*Ease of identifying the healthy food option* was measured by asking how easy it was to identify the healthy product during grocery shopping. Answer options were on a 7-point Likert scale ranging from “1=totally not easy” to “7 = totally easy” [51]. Items to measure *situational motivation to choose healthily* were adopted from Siemsen, Roth, and Balasubramanian [52] and were translated into health-related items. The four items used for “When being presented with the product choices in this particular version of an online store…” were “I had the intention to choose a healthy product,” “I was motivated to choose a healthy product,” “I really wanted to choose a healthy product,” and “I meant to choose a healthy product.” Answer options were on a 7-point Likert scale ranging from “1 = totally disagree” to “7 = totally agree”. The mean of these four items was used as an indication for situational motivation to choose healthily (α =.935).

#### Moderators

*Health interest* was measured with four items developed by Pieniak, Verbeke, Olsen, Hansen, and Brunso [53]. For “In general in my daily life…”; these four items were “health is very important to me,” “I care a lot about my health,” “health means a lot to me,” and “I am very concerned about the health-related consequences of what I do.” Health interest was measured on a 7-point scale ranging from “1 = totally disagree” to “7 = totally agree”. The mean was used as an indication for health interest (α =.921).

#### Background variables

There were also contextual factors that could impact the NP score of consumer’s product choices. Demographics, age, gender, education, and household composition were measured to control for significant differences between conditions. Furthermore, previous label use and familiarity with the Nutri-Score label could influence food choice because the occurrence of previous label use might influence the effect of nutrition labeling on healthy food choices [54]. Previous label use was tested with the statement “I usually compare labels to select the most nutritious food,” rated on a scale ranging from “1 = totally disagree” to “7 = totally agree” [55], and respondents were asked to rate how familiar they were with Nutri-Score labeling (“1=totally not familiar” to “7 = totally familiar”).

Several studies on food swaps investigated acceptability of swap interventions (e.g. [3, 6]). To test the difference in acceptability between swap conditions, i.e., normal swap, swap in combination with Nutri-Score, and/or descriptive norm message, participants in conditions with a swap offer were asked, “You were just offered a healthier food alternative to the food you originally chose. Is this something you would like to have when you do your usual shopping?” [6]. Furthermore, credibility of swap offer(s) was measured with five items by Meyer in [56]: “The alternative product choice that was just offered to me (1) is fair, (2) is unbiased, (3) tells the whole story, (4) is accurate, and (5) is trustworthy.” The mean of these five items was used as indication for credibility of swap offer(s) (α =.886). Both acceptability and credibility were measured on a 7-point scale ranging from “1 = totally disagree” to “7 = totally agree”.

To control for significant differences between groups, frequency of (online) grocery shopping was measured by asking respondents to indicate how often they shop for groceries in a physical store and online supermarket [57], both before and during COVID-19. These four questions were measured on a 7-point scale ranging from “never” to “daily”: (1) never, (2) once a month or less, (3) 2 to 3 times a month, (4) once a week, (5) 2 to 3 times a week, (6) 4 to 5 times a week, or (7) daily. Moreover, to measure whether the simulated online supermarket was perceived as a realistic online shopping experience (credibility of online shopping environment), respondents received the question, “In your opinion, how realistic was the online shopping experience?” They were asked to rate this question on a scale ranging from “1=totally not realistic” to “7 = totally realistic”.

#### Awareness check

Participants were asked whether they had seen 1) a Nutri-Score label, 2) an additional informative message about the product choice of other Dutch consumers, and 3) an alternative product suggestion for a healthier product option after making a product choice. Answer options included “yes”, “no”, and “I do not know”.

### Procedure

After providing informed consent, participants were shown questions to screen for age (older than 18), gender, and country of residence (the Netherlands). After that, eligible participants were shown the following message:

*“Imagine you are at home, figuring out if you still have enough groceries in stock. You find out you need to buy a product from each of the following categories: breakfast cereals, crackers, muesli bars, and pizza. You decide to order these products online.”*

After reading this scenario, respondents were randomly assigned to one of eight study conditions. Randomization was on a 1:1:1:1:1:1:1:1 basis, using computerized random number generation. Intervention allocation was concealed from researchers until after completion of the experiment, as participants were automatically randomized without human involvement. For each condition, four separate questions were shown with the text (*“Imagine you need to buy [breakfast cereals, crackers, muesli bars, pizza] in an online supermarket. Which product would you choose?”*), and the different product options were shown. Participants could choose one product for each question. After that, questions regarding mediators, moderators, and background variables were asked.

### Statistical analysis

Randomization of conditions was checked in terms of demographics and background variables between conditions. An overview of the different conditions is given in Table 1. Descriptive analyses were performed. To assess whether there were significant differences between conditions for age, health interest, previous label use, Nutri-Score familiarity, and credibility of the online shopping environment, a one-way ANOVA was conducted. Pearson Chi Square was used to test for differences in gender, education, household composition, and grocery shopping frequency across conditions. Except for Nutri-Score familiarity, there were no significant differences in demographics and background variables across conditions.

The awareness check tested whether participants in the treatment conditions had noticed the interventions. For each intervention, the frequencies for “yes”, “no”, and “I do not know” were obtained. To analyze the differences in combined NP score across conditions, a one-way ANOVA with Tukey post hoc was conducted for NP values for products combined across conditions. As Levene’s test was highly significant (*p*- values <.01), equal variances could not be assumed. Consequently, Games-Howell post hoc tests and Welch ANOVA test were used to test for hypotheses significance. Then, a 3-way ANOVA with Nutri-Score, swap offer and norm message as independent variables was performed to test the direct and interaction effects of the three interventions on the combined NP score (H1).

To explore the mediating effect of ease of identifying the healthy food option and situational motivation to choose healthily between the three interventions and the dependent variable combined NP score, while also taking into account the moderating effect of health interest and situational motivation, PROCESS by Hayes [58] was used. PROCESS Model 21 was used to explore the effect of Nutri-Score and swap offer on the combined NP score via the ease of identifying the healthy food option (H2/H4/H8), while also testing for moderators health interest (H10) and situational motivation (H9) (moderated moderated mediation). To explore the effect of Nutri-Score labeling and swap offer on the combined NP score via situational motivation to choose healthily (H3/H5/H7), while also considering the moderating effect of health interest (H11), PROCESS Model 7 was used (moderated mediation). All data was analyzed using IBM SPSS Statistics 24 with a significance level of *p* < .05.

## Results

### Descriptives and randomization check

This research was conducted among 550 participants divided over eight conditions (see Table 2). Gender was equally balanced across conditions, and the average age was 45.12 (SD = 16.45). All 550 participants lived in the Netherlands. An overview of the sociodemographic characteristics age, gender, and education is given in Table 2, and an overview for household composition and frequency of (online) grocery shopping across conditions is shown in Additional file 7.

**Table 2:** Demographics of the sample across conditions

	Total sample *N* = 550	Condition 1) Control *N* = 69	Condition 2) Nutri-Score *N* = 69	Condition 3) Norm message *N* = 69	Condition 4) Swap offer *N* = 67	Condition 5) Nutri-Score + message *N* = 69	Condition 6) Nutri-Score + swap *N* = 69	Condition 7) message + swap *N* = 69	Condition 8) All three *N* = 69	***p***-value
Gender										
Male	275	32	32	39	31	32	30	38	41	0.446 ^1,3^
Female	274	36	37	30	36	37	39	31	28	
Other	1	1	0	0	0	0	0	0	0	
Age (M, SD)	45.12 (16.45)	46.52 (16.89)	42.75 (15.68)	44.49 (16.71)	45.54 (15.46)	43.64 (16.80)	45.54 (16.38)	42.93 (15.99)	49.57 (17.36)	0.228 ^2^
Education										
Elementary school or low secondary education	106	17	9	10	13	16	14	11	16	0.490 ^1^
High school or secondary education	242	32	30	28	29	31	32	26	34	
Higher education or university	202	20	30	31	25	22	23	32	19	

^1^ X^2^ test (Chi Square)

^2^ One-way ANOVA, F-test

^3^ X^2^ test performed without category “other”

#### Randomization and awareness checks

##### Randomization checks

There were no differences across the eight conditions at a 5% significance level in gender (X^2^ (7) = 6.839, *p* = .45), age (F(7,542) = 1.28, *p* = .26) and educational level (X^2^ (14) = 13.464, *p* = .49), as shown in Table 2. Also, no significant differences were found for household composition (X^2^ (21) = 23.244, *p* = .33), physical grocery shopping frequency before (X^2^ (28) = 34.424, *p* = .19) and during COVID-19 (X^2^ (21) = 17.633, *p* = .67), online grocery shopping frequency before (X^2^ (14) = 10.567, *p* = .72) and during COVID-19 (X^2^ (14) = 11.301, *p* = .66), previous label use (F(2, 542) = 1.39, *p* = .21), and average health interest (F(7, 542) = 0.37, *p* = .97) (Additional file 7). This means that randomization was successful. There was a significant difference for Nutri-Score familiarity (F(7, 542) = 2.79, *p* < .01) between the Nutri-Score (condition 2) and swap offer condition (condition 4). Moreover, respondents in the conditions with a swap offer were asked about their swap acceptability and perceived credibility. As for *swap acceptability*, 54% of the respondents who received a swap somewhat to fully agreed with wanting to receive a swap offer during usual online grocery shopping, and there were no significant differences across the four conditions with a swap (F(3,255) = .09, *p* = .97). More than half of the respondents wanted a swap offer, but *credibility* of the current studies’ swaps had a mean value of only 4.53 (SD = 1.11). Most respondents (41.5%) were neutral about the swap credibility, and there were no significant differences across the four swap conditions (F(3,254) = .79, *p* = .50).

##### Awareness checks

To check whether participants noticed interventions, a crosstabulation with Chi Square was performed (Table 3). A cross-tabulation with swap offer (X^2^ (2, *N* = 550) = 51.07, *p* < .001), Nutri-Score label (X^2^ (2, *N* = 550) = 128.150, *p* < .001), and descriptive norm message (X^2^ (2, *N* = 550) = 20.95, *p* < .001) was significant, illustrating that the proportions of the answer options depended on the intervention being present or not.

**Table 3:** Awareness check for the three interventions (swap offer, Nutri-Score, descriptive message)

	Answer options			
	Yes	No	I do not know	Total
*Noticed swap offer*				
No swap offer (condition 1, 2, 3, 5)	64	146	66	276
With swap offer (condition 4, 6, 7, 8)	144	95	35	274
*Noticed Nutri-Score*				
No Nutri-Score (condition 1, 3, 4, 7)	64	132	48	274
With Nutri-Score (condition 2, 5, 6, 8)	196	53	27	276
*Noticed social norm message*				
No message (condition 1, 2, 4, 6)	66	154	54	274
With message (condition 3, 5, 7, 8)	116	111	49	276

*Note*. All *p*-values for X^2^ test are < 0.001

#### Dependent variable and mediators

Table 4 shows means and standard deviations for the dependent variable and the mediators for the total sample and across conditions involving all participants who were randomly assigned. Half of the respondents (50.73%) found it easy to identify healthy food options. *Ease of identifying the healthy food option* was lowest in the swap condition (M = 3.72, SD = 1.69), while the highest mean score was found for the condition with all three interventions (M = 5.06, SD = 1.57). These differences were significant as determined by a one-way ANOVA, F(7,542) = 5.87, *p* < .001. Results showed that *situational motivation to choose healthily* was present for most respondents, and there were no significant differences across conditions (F(7,542) = .89, *p* = .52).

**Table 4:** Dependent variable and mediators across conditions

		Nutri-Score (*n* = 276)				No Nutri-Score (*n* = 274)				
		Swap (*n* = 138)		No Swap (*n* = 138)		Swap (*n* = 136)		No Swap (*n* = 138)		
	Total Sample (*n* = 550)	Message (*n* = 69)	No Message (*n* = 69)	Message (*n* = 69)	No Message (*n* = 69)	Message (*n* = 69)	No Message (*n* = 67)	Message (*n* = 69)	No Message (*n* = 69)	*p*-value^1^
Situational motivation (M, SD)	4.54 (1.52)	4.54^a^ (1.37)	4.64^a^ (1.48)	4.70^a^ (1.49)	4.59^a^ (1.69)	4.41^a^ (1.64)	4.29^a^ (1.48)	4.67^a^ (1.49)	4.26^a^ (1.52)	.52
Ease of identifying (M, SD)	4.46 (1.70)	5.06^d^ (1.57)	4.80^bcd^ (1.59)	4.93^cd^ (1.65)	4.72^bcd^ (1.72)	4.04^ab^ (1.82)	3.72^a^ (1.69)	4.26^abcd^ (1.59)	4.13^abc^ (1.55)	<.001
Combined NP score (M, SD)	11.57 (15.44)	5.14^a^ (12.70)	5.13^a^ (12.97)	14.06^ab^ (17.02)	15.38^b^ (15.24)	6.35^a^ (13.23)	10.52^b^ (16.15)	17.96^b^ (15.15)	18.03^b^ (14.02)	<.001

*Note*. Means in a row sharing the same superscript are not significantly different from each other (*p* < .05) according to Tukey post hoc test

^1^ One-way ANOVA, F-test

**Fig. 3:**
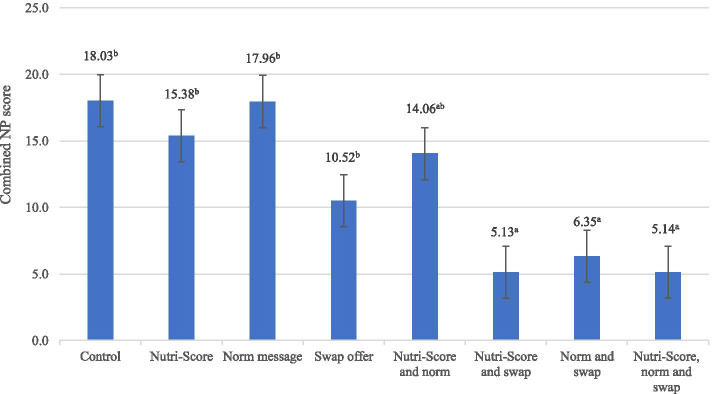
Mean values and SE of the combined NP score for each condition. *Note*. Means sharing the same superscript are not significantly different from each other (*p* < .05) according to Games-Howell post hoc test

A one-way ANOVA was conducted to compare combined NP score across conditions (Table 4). Results showed statistically significant differences in the combined NP score (F(7,542) = 9.86, *p* < .001). Diving deeper into the effect of conditions on the combined score (Fig. 3) with a highly significant Levene’s test statistic (F(7,542) = 3.04, *p* < .01), Welch’s ANOVA test revealed that the eight conditions significantly differed in combined NP score, F(7, 232.017) = 10.82, *p* < .001. The Games-Howell post hoc test showed a statistically significant difference only between certain conditions (see Fig. 3).

### Hypotheses testing

#### Effect of interventions on combined NP score (hypothesis 1)

To test the primary hypothesis, a 3-way ANOVA was performed with interventions as independent variables and combined NP score as dependent variable (H1). Simple main effects showed that combined NP score was indeed significantly influenced by Nutri-Score labeling (F(1,542) = 6.93, *p* < .01) and swap offer (F(1,542) = 58.74, *p* < .001), while the effect of norm message was not significant (F(1,542) = 1.24, *p* = .27). No statistically significant 2-way and 3-way interaction were found, as shown with F-test statistics in Table 5. Therefore, parameter estimate results were calculated with only the direct effect of interventions on the dependent variable and showed that presenting a Nutri-Score label and offering a swap decreased the combined NP score with 3.276 (*p* < .001) and 9.579 (*p <* .001), respectively (Table 6).

**Table 5:** 2 (Nutri-Score) × 2 (norm message) × 2 (swap offer) ANOVA with combined NP score as dependent variable

Factor	df	F	***p***	Ƞ^**2**^
**Nutri-Score**	1	6.929	.009	.013
**Norm message**	1	1.236	.267	.002
**Swap offer**	1	58.743	.000	.098
Nutri-score*Norm message	1	.347	.556	.001
Nutri-Score*Swap offer	1	.000	.993	.000
Norm message*Swap offer	1	.307	.580	.001
Nutri-Score*Norm message*Swap offer	1	1.185	.277	.002
**df**_**ERROR**_	542

**Table 6:** Regression parameter estimates results: direct effect of interventions on the combined NP score

Factor	Estimate	SE	***p***	CI		Ƞ^**2**^
				LB	UB	
**Intercept**	18.682	1.25	.000	16.234	21.129	.292
**Nutri-Score**	−3.276	1.25	.009	−5.724	−.829	.013
**Norm message**	−1.378	1.25	.269	−3.825	1.070	.002
**Swap offer**	−9.579	1.25	.000	−12.026	−7.132	.098

#### Mediation and moderation analysis

In addition to the primary analysis, the underlying process was explored with ease of identifying the healthy food options and situational motivation to choose healthily as mediators and health interest as moderator.

##### Direct effect of interventions on combined NP score (H1)

The direct effect of both swap offer and Nutri-Score on combined NP score were significant with both ease of identification (b = −10.01, *p* < .001 and b = −2.60, *p* = .04 respectively) and situational motivation (b = − 9.89, *p* < .001 and b = − 2.62, *p* = .03 respectively) as mediators.

##### Swap offer on ease of identification (H2) and situational motivation (H3)

Mediation analysis did not find evidence of a significant effect of swap offer on the ease of identifying the healthy food option (*b* = 0.69, *p* = .28), nor on the situational motivation to choose healthily (*b* = .44, *p* = .38).

##### Nutri-score on ease of identification (H4) and situational motivation (H5)

We found no evidence of an effect between Nutri-Score and ease of identifying the healthy food option (*b* = −.40, *p* = .52) as well as between Nutri-Score and situational motivation to choose healthily (*b* = −.19, *p* = .71).

##### Situational motivation on combined NP score (H7)

The effect of situational motivation on combined NP score was tested with both swap offer and Nutri-Score as independent variable. The effect was significant for both swap offer (b = − 3.40, *p* < .001) and Nutri-Score (*b* = − 3.25, *p* < .001).

##### Ease of identification on combined NP score (H8)

The ease of identifying the healthy food option on NP score was non-significant for both swap offer (*b* = .88, *p* = .37) and Nutri-Score (*b* = .50, *p* = .63).

##### Effect of situational motivation to choose healthily on association between ease of identification and combined NP score (H9)

With swap offer as independent variable, situational motivation did not significantly moderate the relationship between ease of identifying the healthy food option and combined NP score (*b* = −.24, *p* = .21), and for the moderating effect, no evidence was found of a significant effect when Nutri-Score was the independent variable (*b* = −.10, *p* = .62).

##### Effect of moderator health interest on association between interventions and ease of identifying the healthy food option (H10a and H10b)

With regard to moderator health interest (H10), a significant effect was found for the moderating effect on the relationship between Nutri-Score and ease of identifying the healthy food option (b = .23, *p* = .04) (H10b). No evidence was found for a significant effect with a swap offer as intervention (b = −.16, *p* = .19) (H10a).

##### Effect of moderator health interest on association between interventions and situational motivation (H11a and H11b)

When testing the effect of the moderator health interest on the effect between interventions and situational motivation, no evidence was found for a significant relationship between swap offer and situational motivation as proposed in hypothesis 11a (*b* = −.12, *p* = .22). Also, no evidence for the effect proposed in hypothesis 11b of the moderator health interest on the relationship between Nutri-Score and situational motivation was found (*b* = .07, *p* = .44).

##### Total effects

**Fig. 4:**
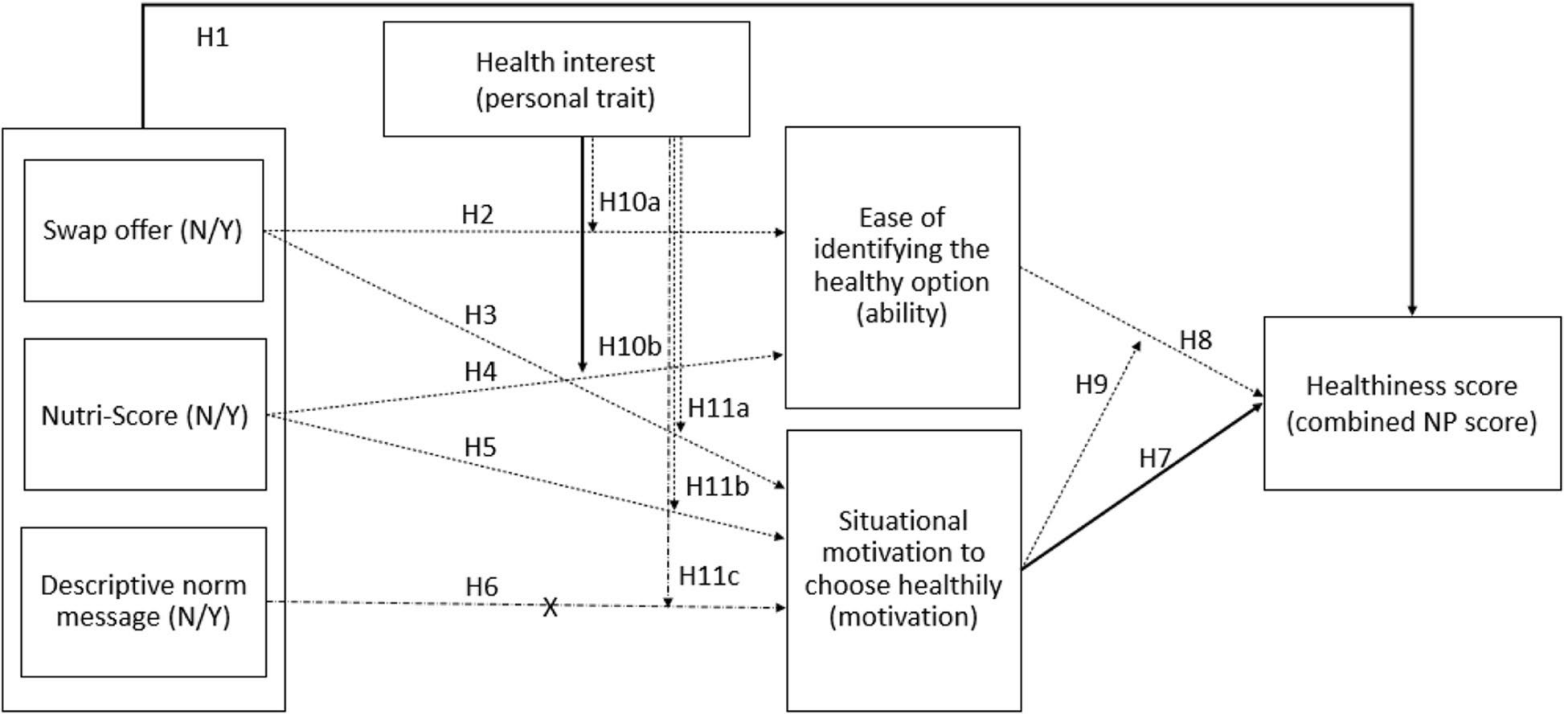
Theoretical framework with significant effects (straight lines), insignificant effects (dotted line), and potential effects that were not tested because of insignificant main effect (dotted line with X)

PROCESS Model 21 was also used to test the moderated moderated mediation effect of the model, which quantifies the indirect effect of swap offer (IV1) or Nutri-Score (IV2) on combined NP score (DV) through ease of identification (M) while taking into account the moderating effect of both health interest and situational motivation. No evidence for a significant effect was found for swap offer (Index = .0382, bootstrapped 95% CI: −.0384 to.1484) or for Nutri-Score (Index = −.0240, bootstrapped 95% CI: −.1543 to.0778). As for the moderated mediation effect tested with PROCESS Model 7, which quantifies the effect of health interest (moderator) on the indirect effect of Nutri-Score labeling (IV) on combined NP score (DV) through situational motivation (M), we found no evidence for significant relationships. (Index = −.2380, bootstrapped 95% CI: −.9127 to.4143). Full results of primary and explorative analyses are provided in Fig. 4.

## General discussion

### General discussion

There are many types of interventions that can shape consumer decision-making in the online grocery shopping environment. Online grocery shopping environments can offer novel and more efficient ways for stimulating healthy food choices. The present study examined the effect of swap offers. Nutri-Score labeling, and a norm message on the NP score of food choices in an online grocery store environment. Additionally, we explored whether the effect of the interventions on the combined NP score of food choices is mediated by ease of identification and situational motivation to choose healthily, while also exploring the moderating effect of health interest. In our simulated online grocery shopping environment, participants were asked to choose a product from an assortment of six types of breakfast cereals, crackers, muesli bars, and pizzas.

Current results on the primary analysis provided evidence that Nutri-Score labeling and offering an alternative healthier product with a food swap can be used to guide people toward healthier food choices. Nonetheless, results did not provide any evidence that interventions have a synergistic effect on the NP score when combined. Findings of the current study confirmed that Nutri-Score labeling and swap offers lead to a better combined NP score, which is in line with previous findings (e.g. [4, 20, 59]). Compared to the control condition, the combined NP score decreased by 16.6% (from 18.03 to 15.38) when a Nutri-Score label was displayed and by 41.7% (from 18.03 to 10.52) when consumers were offered an alternative product option. A positive effect of Nutri-Score [18–20, 28] and swap offers [7–9] on the NP score of chosen products is in line with several other studies.

However, norm messages did not impact participants’ food choices. This finding contradicts a common finding of behavioral research (e.g., [60]) but may be explained by the notion that a descriptive norm message effect is greater when the behavior is public versus private [61]. Additionally, the norm message did not specifically guide consumers to a healthier option, so it might have been difficult for consumers to detect the healthiest food option when only a norm message was provided. Also, the norm message was noticed by less than half of the participants in norm message conditions, which could have affected results. Future studies could use a more salient norm message to test its effect on NP score. For the swap offer, around one-third of the respondents in swap conditions could not confirm or were unsure about having received a swap offer, and the same was true for around a quarter of Nutri-Score condition respondents. This could be because respondents forgot or were unaware of the swap or because the survey question used in the awareness check was not well understood. Future research could explore how such interventions are processed by consumers.

With regard to the processes behind the effect of interventions, our exploratory results showed that, contrary to expectations, interventions did not make healthier options easier to identify. This might be because our interventions were unfamiliar to the participants or did not draw sufficient attention to change purchase behavior [62]. Recent studies (e.g., [19, 20]) suggested that Nutri-Score can increase consumers’ ability to better understand the nutritional value of products, and therefore identifying which food is healthier becomes easier. Potential reasons for insignificant effects in this study are that respondents did not understand or believe the Nutri-Score label, as 236 (42.91%) respondents were not familiar with this label, or that no additional nutrition information about the product was given. The need for additional information alongside a Nutri-Score label was shown by Julia et al. [22], who showed that Nutri-Score labels are only effective when combined with an educational leaflet.

Participants who got the swap recommendation were specifically told ‘this product is healthier’ to make the healthfulness of the selected and recommended product fully transparent, which indeed led to healthier food choices. However, this explicit text did not make identifying healthy food products easier. A potential reason for insignificant results of this mediating variable might be question ambiguity. Respondents might have thought that they had to rate easiness of identifying the healthier food options without considering the swap offer, instead of including the swap offer. In addition, future research should measure ease of identification with multiple items, instead of a single item.

Situational motivation to choose healthily also did not mediate the effect between interventions and combined NP score, most likely because extrinsic incentives alone do not increase situational motivation to choose healthily [63, 64]. Extrinsic incentives (i.e., Nutri-Score label or swap offer) often only work if intrinsic motivation to perform a certain behavior is also present [63], so a personal goal should provide a behavioral benchmark to follow extrinsic incentives [64]. Specifically for Nutri-Score labeling, its effect on combined NP score of food choices might not be increased by situational motivation because knowledge about Nutri-Score label was low. Only about 40% of participants indicated knowledge of the Nutri-Score label, and therefore this label might not have increased health motivation at the moment of choice. Results may be different once Dutch consumers will become more familiar with Nutri-Score. This can be investigated by conducting research in other countries such as Belgium, where inhabitants are already more familiar with Nutri-Score labeling, or future studies in the Netherlands when the Nutri-Score will have been present longer. Moreover, future research can be conducted to test whether interventions have a long term effect on motivation leading to positive effects in future purchases instead of only in this particular shopping trip. Future studies are also advised to investigate the process more in depth and identify the mediators.

In the current paper, health interest was expected to moderate the relationship between interventions and mediators. This was based on earlier findings that the goal of interventions should be in line with personal goals of eating healthy [44, 45, 65]. Consumers with higher health motivations were not more susceptible to the interventions than consumers with lower health motivation. Health interest only moderated the relationship between Nutri-Score and ease of identification, implying that Nutri-Score labeling worked better to identify healthy food options when consumers were interested in healthy eating.

### Limitations and future studies

Future studies should also address some of the limitations inherent in this study. General limitations are the limited number of products used in the experiment and to what extent the shopping environment was perceived as realistic. Respondents were not actually going to buy groceries, so results are about intentions rather than actual behavior. A field experiment using an actual online supermarket with a broad product range can refine insights on the effectiveness of interventions on actual purchases. Even though we omitted brand and price information from the product packaging, the products might still be recognized by the Dutch participants, which may negatively impact the external validity.

Additionally, familiarity with the Nutri-Score label was higher in some experimental conditions, which may have created a confounding effect in this study. Future studies can also investigate whether the effect of Nutri-Score labeling changes when consumers have been exposed to nutritional labels on the local market and are likely to have a higher awareness. Furthermore, Nutri-Score received some criticism, as it does not (yet) fully align with all local nutritional recommendations in the countries where it is applied, which begs for further investigation on the effect of Nutri-Score [66]. As a result, Nutri-Score’s international steering committee is reviewing and proposing changes to further improve the Nutri-Score algorithm and improve its alignment with the national nutritional and dietary recommendations [17].

Finally, a potential reason for not finding many significant effects for mediating and moderating associations is that we asked participants questions regarding these effects after the experiment of choosing food options (temporal order) or because we did not have enough statistical power to explore these hypotheses. We performed our power analysis for the primary analysis (H1) and explorative hypotheses were tested without any additional power calculation. This means that results for explorative hypotheses might be less conclusive. We encourage future researchers to conduct follow-up experiments that investigate what motivates consumers to select a healthy option which is also easy to identify.

### Conclusion and practical implications

In sum, our results provide evidence that Nutri-Score and swap offers can be used in the online store environment to guide people toward healthier food choices. We did not find evidence that the effects of Nutri-Score and food swaps are mediated through situational motivation to choose healthily and ease of identifying healthy food options. Our results have several practical implications. First, the findings indicate that retailers can use swap offers to help people choose healthier products. Care should be taken that the swap offer is appealing and offers a credible alternative to the first choice of consumers, for example by explaining the health reason behind the swap. Second, results provide evidence that Nutri-Score labeling can guide consumers toward healthier food choices in an online store environment. Thus, retailers could use Nutri-Score labeling in the online store environment to steer consumers. It should be noted that the results indicate the need to increase awareness of Nutri-Score labeling among Dutch consumers. Currently, knowledge of the Nutri-Score label is quite low and therefore communication campaigns about the label are advised after introducing the label in the Netherlands in 2021. Moreover, it is important for practitioners to keep in mind that Nutri-Score labeling is most likely to be effective when all products in the online environment are displayed with a Nutri-Score label [13]. This way, consumers are best able to make healthier food choices. Hence, swap offers and Nutri-Score labeling are promising methods to guide consumers to healthier food choices in the online grocery store.

## Supplementary Information

**Additional file 1.** CONSORT checklist. Description of data: Additional file 1 is a checklist of information to include when reporting a randomized trial.

**Additional file 2.** TIDieR checklist. Description of data: Additional file 2 is a checklist for intervention description and replication.

**Additional file 3.** Overview of the images and products used for each product category. Description of data: This additional file gives an overview of the product images that were used in the survey for each product category. The file consists of four categories: (A) breakfast cereals, (B) Crackers, (C) Muesli bars, and (D) Pizza, each consisting of six products.

**Additional file 4.** Explanation of calculation Nutri-Score. Description of data: Additional file 4 is a table with the calculation for the NP score and corresponding Nutri-Score label for each product that was used in the survey.

**Additional file 5.** Examples of product choices with a (1) swap offer, (2) Nutri-Score, or (3) descriptive norm message. Description of data: Additional file 5 shows examples of the survey questions that were displayed for the swap offer (Figure A5.1), Nutri-Score labeling (Figure A5.2), and descriptive norm message condition (Figure A5.3).

**Additional file 6.** Overview of exposures in each treatment group. Description of data: This additional provides an overview of the exposures in each condition. A table is provided where each condition is separately explained.

**Additional file 7.** Characteristics of the sample across conditions. Description of data: Additional file 7 provides a table with the characteristics of the sample for each condition. Variables presented are household composition, grocery shopping frequency separately for off-line, and online grocery shopping, as well as before and during COVID-19, previous label use, familiarity Nutri-Score, health interest, credibility swap and acceptability.

## Data Availability

The data set supporting the conclusions of this article is available in the Zenodo repository, [10.5281/zenodo.4309693].
