# Supplementary material for: The use of food swaps to encourage healthier online food choices: a randomized controlled trial
Source: Int J Behav Nutr Phys Act. 2021 Dec 4;18:156. doi: 10.1186/s12966-021-01222-8 (PMC8642761; doi:10.1186/s12966-021-01222-8)
Supplement: Supplementary file 3 — Additional file 3. Overview of the images and products used for each product category. Description of data: This additional file gives an overview of the product images that were used in the survey for each product category. The file consists of four categories: (A) breakfast cereals, (B) Crackers, (C) Muesli bars, and (D) Pizza, each consisting of six products. [file 12966_2021_1222_MOESM3_ESM.pdf]

Additional file 3. Overview of the images and products used for each product category

Category A) Breakfast cereals

	Product 1	Product 2	Product 3	Product 4	Product 5	Product 6
**Image**	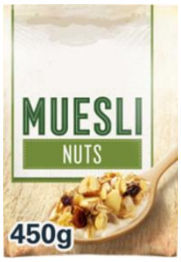	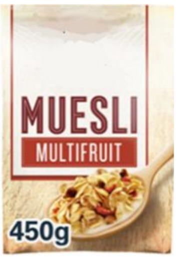	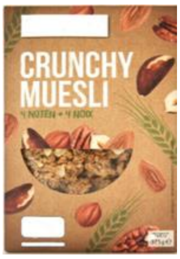	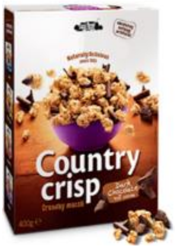	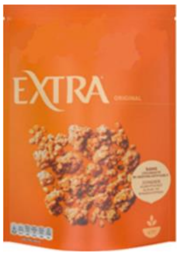	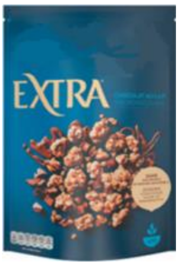

Category B) Crackers

	Product 1	Product 2	Product 3	Product 4	Product 5	Product 6
**Image**	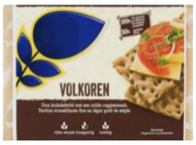	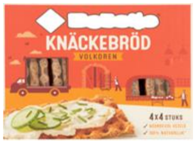	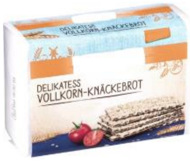	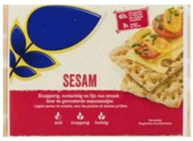	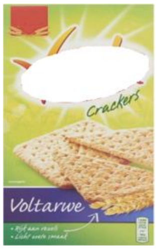	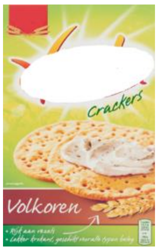

Category C) Muesli bar

	Product 1	Product 2	Product 3	Product 4	Product 5	Product 6
**Image**	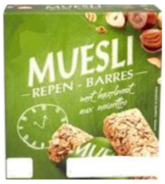	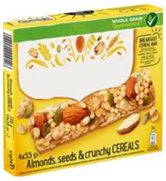	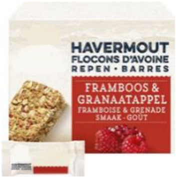	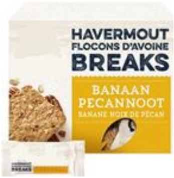	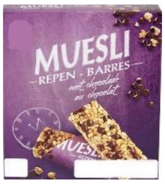	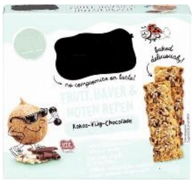

Category D) Pizza

	Product 1	Product 2	Product 3	Product 4	Product 5	Product 6
**Image**	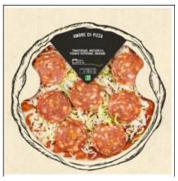	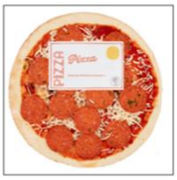	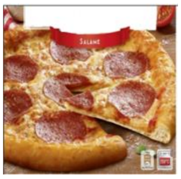	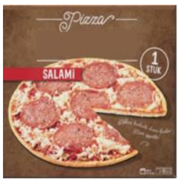	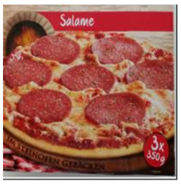	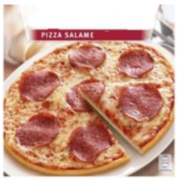
