# Supplementary material for: The use of food swaps to encourage healthier online food choices: a randomized controlled trial
Source: Int J Behav Nutr Phys Act. 2021 Dec 4;18:156. doi: 10.1186/s12966-021-01222-8 (PMC8642761; doi:10.1186/s12966-021-01222-8)
Supplement: Supplementary file 4 — Additional file 4. Explanation of calculation Nutri-Score. Description of data: Additional file 4 is a table with the calculation for the NP score and corresponding Nutri-Score label for each product that was used in the survey. [file 12966_2021_1222_MOESM4_ESM.pdf]

Additional file 4. Calculation of NP score and Nutri-Score category

Table A4. Nutri-Score values (Ofcom/NP score, and corresponding Nutri-score category) for each product. (Calculation: Excel Sheet Santé Publique France (2020))

Product type	Product	Total fat g/100g or 100mL	Saturated fat g/100g or 100mL	Sugars g/100g or 100mL	Proteins g/100g or 100mL	Salt g/100g or 100mL	Fibres g/100g or 100mL	Sodium mg/100g or 100mL	NP score	Nutri-Score
**Breakfast cereals**	Product 1*	8,7	1,3	4,8	11	0,06	9	24	-4	Nutriscore_A
	Product 2	7,3	1,1	4,9	10	0,05	9,4	20	-4	Nutriscore_A
	Product 3	22	3,4	10	10	0,14	13	56	0	Nutriscore_B
	Product 4	16,7	5,7	20,7	8,9	0,03	5,9	12	9	Nutriscore_C
	Product 5	22	11	19	8	0,8	5,7	320	18	Nutriscore_D
	Product 6	22	12	25	7	0,68	4,9	272	19	Nutriscore_E
**Pizza**	Product 1*	7,5	3	2	9,5	1,03	1,5	412	2	Nutriscore_B
	Product 2	8,5	3,9	2,6	11,1	1,08	2,1	432	2	Nutriscore_B
	Product 3	8,8	4,6	3,3	9,5	1,5	2,07	600	10	Nutriscore_C
	Product 4	14	4,5	4	11	1,18	2	472	10	Nutriscore_C
	Product 5	9,5	4,4	3,4	10,5	1,15	0	460	11	Nutriscore_D
	Product 6	14	4,8	3,1	10	1,4	2	560	11	Nutriscore_D
**Crackers**	Product 1*	2	0,4	1,5	9	1	20	400	-2	Nutriscore_A
	Product 2	7,7	0,7	1,8	12	0,99	12	396	-1	Nutriscore_A
	Product 3	1,8	0,3	2,2	8,4	1,5	16	600	0	Nutriscore_B
	Product 4	10	1,5	1,5	13,8	1	9	400	0	Nutriscore_B
	Product 5	6,7	2,2	18	9,9	0,65	8,5	260	7	Nutriscore_C
	Product 6	12	5,1	2,1	11,5	1,88	8	752	13	Nutriscore_D
**Muesli bar**	Product 1*	13	2,6	11	8,1	0,05	25	20	-2	Nutriscore_A
	Product 2	18,1	2	16,5	11,2	0,4	7,5	160	0	Nutriscore_B
	Product 3	10	1,3	18	7,1	0,45	9,1	180	1	Nutriscore_B
	Product 4	17	2,8	12	7,8	0,61	6,1	244	6	Nutriscore_C
	Product 5	15	7,2	24	5,8	0,6	5	240	14	Nutriscore_D
	Product 6	30	22	25	9	0,1	9,5	40	15	Nutriscore_D

*This product was offered in the swap
