# Supplementary material for: The use of food swaps to encourage healthier online food choices: a randomized controlled trial
Source: Int J Behav Nutr Phys Act. 2021 Dec 4;18:156. doi: 10.1186/s12966-021-01222-8 (PMC8642761; doi:10.1186/s12966-021-01222-8)
Supplement: Supplementary file 5 — Additional file 5. Examples of product choices with a (1) swap offer, (2) Nutri-Score, or (3) descriptive norm message. Description of data: Additional file 5 shows examples of the survey questions that were displayed for the swap offer (Figure A5.1), Nutri-Score labeling (Figure A5.2), and descriptive norm message condition (Figure A5.3). [file 12966_2021_1222_MOESM5_ESM.pdf]

Additional file 5. Examples of product choices with a (1) Nutri-Score, (2) swap offer, or (3) descriptive norm message.

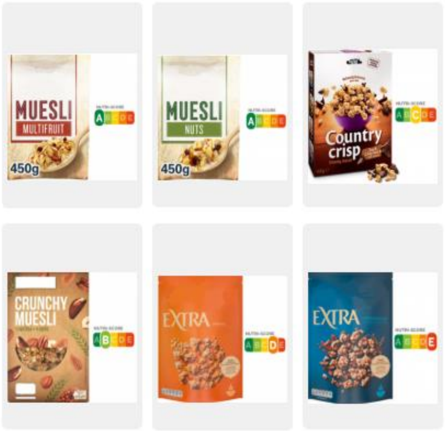

Figure A5.1. Example of product choices with a Nutri-Score label. Corresponding text: *"Imagine you are going to buy cereal in an online supermarket, which product would you choose?"*.

You chose: 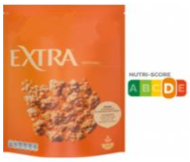

(1) 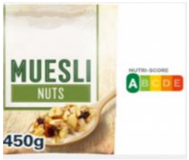

(2) 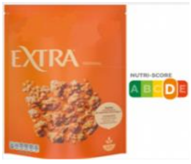

Figure A5.2. Example of product choices with a swap offer (including Nutri-Score). Corresponding text: *"You chose [chosen option]. A better choice would be to choose a healthier product such as the product displayed below. Which product do you choose?"*. Answer options: *"(1) the healthier choice or (2) your first choice"*.

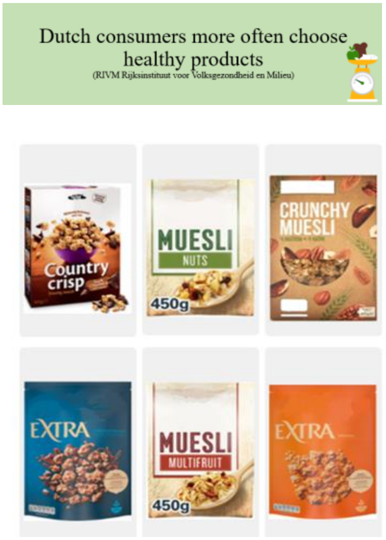

Figure A5.3. Example of product choices with a descriptive norm message. Corresponding text: *"Imagine you are going to buy cereal in an online supermarket, which product would you choose?"*.
