# Supplementary material for: The use of food swaps to encourage healthier online food choices: a randomized controlled trial
Source: Int J Behav Nutr Phys Act. 2021 Dec 4;18:156. doi: 10.1186/s12966-021-01222-8 (PMC8642761; doi:10.1186/s12966-021-01222-8)
Supplement: Supplementary file 6 — Additional file 6. Overview of exposures in each treatment group. Description of data: This additional provides an overview of the exposures in each condition. A table is provided where each condition is separately explained. [file 12966_2021_1222_MOESM6_ESM.pdf]

Additional file 6. Overview of exposures in each treatment group

Table A6. Exposures in each treatment group

Treatment group	Exposure
1: Control	Participants were shown six product choices for the four categories (2 rows, 3 columns). The four categories with corresponding six products are the same for all treatment groups, but the order of categories and products is randomized for each participant.
2: Nutri-Score	All product choices were shown with a Nutri-Score label on the right side of the product (see Additional file 5 figure A5.1)
3: Norm message	The product choices were shown with one norm message banner above the product choices (see Additional file 5 figure A5.3)
4: Swap offer	First, product options were shown in the same way as the control condition. Then, if a healthier alternative was available for the initial chosen product, a swap was offered immediately. The swap showed the product the participant had chosen and the possibility to stick to that choice or choose a healthier alternative (see Additional file 5 figure A2).
5: Nutri-Score + norm message	All product choices were displayed with a Nutri-Score label on the right side of the product and on top of the product choices a norm message banner was presented.
6: Nutri-Score + swap offer	First, products were shown in the same way as the Nutri-Score condition, but after the product choice was made a swap was offered if a healthier alternative was available. The swap showed the product the participant had chosen and the possibility to stick to that choice or choose a healthier alternative. All product options were shown with a Nutri-Score label.
7: Norm message + swap offer	First, products were shown in the same way as the norm message condition, but after the product choice was made a swap was offered if a healthier alternative was available. The swap showed the product the participant had chosen with a norm message banner on top and the possibility to stick to that choice or choose a healthier alternative.
8: Nutri-Score + norm message + swap offer	A combination of all treatment groups was presented. This means that product choices were shown with a Nutri-Score label alongside each product and a norm message banner on top of the products. After product choices, a swap was offered if a healthier alternative was available. The swap was also displayed with the Nutri-Score label alongside each product and with a banner on top of the swap message.
