# Supplementary material for: The use of food swaps to encourage healthier online food choices: a randomized controlled trial
Source: Int J Behav Nutr Phys Act. 2021 Dec 4;18:156. doi: 10.1186/s12966-021-01222-8 (PMC8642761; doi:10.1186/s12966-021-01222-8)
Supplement: Supplementary file 7 — Additional file 7. Characteristics of the sample across conditions. Description of data: Additional file 7 provides a table with the characteristics of the sample for each condition. Variables presented are household composition, grocery shopping frequency separately for off-line, and online grocery shopping, as well as before and during COVID-19, previous label use, familiarity Nutri-Score, health interest, credibility swap and acceptability. [file 12966_2021_1222_MOESM7_ESM.pdf]

Additional file 7. Characteristics of the sample across conditions

Table A7. Characteristics of the sample across conditions

	**Total sample** N = 550	**Condition 1)** Control N = 69	**Condition 2)** Nutri-Score N = 69	**Condition 3)** Norm message N = 69	**Condition 4)** Swap offer N = 67	**Condition 5)** Nutri-Score + message N = 69	**Condition 6)** Nutri-Score + swap N = 69	**Condition 7)** message + swap N = 69	**Condition 8)** All three N = 69	**p-value**
Household composition										
Single parent with child(ren)	44	11	8	4	2	6	3	3	7	
Single without child(ren)	120	15	17	16	18	17	14	10	13	
Married with child(ren)	188	16	25	25	23	22	29	29	19	0.331 [1,3]
Married without child(ren)	171	24	17	20	20	21	21	23	25	
Living with roommates/friends	24	2	2	4	4	3	2	3	4	
Other…	3	1	0	0	0	0	0	1	1	
Grocery shopping frequency										
Physical store before COVID-19										
Two or three times a month or less	45	3	10	8	7	6	3	5	3	
Once a week	127	11	17	9	18	11	26	16	19	0.187 [1]
Two or three times a week	241	34	25	34	30	32	28	32	26	
Four or five times a week	78	9	10	13	9	9	5	10	13	
Daily	59	12	7	5	5	9	7	6	8	
Online store before COVID-19										
Two or three times a month or less	440	59	56	52	54	53	56	51	59	0.720 [1]
Once a week	61	7	5	9	6	9	9	10	6	
Two or three times a week or more	49	3	8	8	9	5	4	8	4	
Physical store during COVID-19										
Two or three times a month or less	96	11	13	13	14	7	7	19	12	
Once a week	205	24	26	22	24	26	35	21	27	0.672 [1]
Two or three times a week	185	25	21	28	22	25	21	22	21	
Four or five times a week or more	64	9	9	6	9	9	6	7	9	

Table A7 (continued).

	**Total sample** N = 550	**Condition 1)** Control N = 69	**Condition 2)** Nutri-Score N = 69	**Condition 3)** Norm message N = 69	**Condition 4)** Swap offer N = 67	**Condition 5)** Nutri-Score + message N = 69	**Condition 6)** Nutri-Score + swap N = 69	**Condition 7)** message + swap N = 69	**Condition 8)** All three N = 69	**p-value**
Previous label use (M, SD)	4.30 (1.61)	4.12 (1.68)	4.17 (1.86)	4.32 (1.49)	4.54 (1.52)	4.21 (1.49)	4.72 (1.33)	4.26 (1.81)	4.04 (1.59)	0.209[2]
Familiarity Nutri-Score (M, SD)	3.66 (1.82)	3.49 (1.72)	4.25 (1.78)	3.41 (1.75)	3.18 (1.76)	4.03 (1.83)	3.88 (1.75)	3.41 (2.02)	3.61 (1.74)	0.007[2]
Health interest (M, SD)	5.28 (1.21)	5.19 (1.24)	5.19 (1.33)	5.25 (1.11)	5.31 (1.09)	5.21 (1.17)	5.39 (1.22)	5.31 (1.16)	5.35 (1.35)	0.973[2]
Credibility swap (M, SD)	4.53 (1.11)	-[4]	-[4]	-[4]	4.38 (1.04)	-[4]	4.62 (0.89)	4.48 (1.23)	4.64 (1.23)	0.500[2]
Acceptability swap (M, SD)	4.55 (1.61)	-[4]	-[4]	-[4]	4.50 (1.54)	-[4]	4.51 (1.56)	4.63 (1.86)	4.55 (1.46)	0.968[2]

[1] χ^2 test
[2] One way ANOVA, F-test
[3] χ^2 test performed without category "other" and "living with roommates/friends"
[4] Questions about credibility and acceptability of the swap were not asked in these conditions
